# Predictors of Frailty in Patients With Liver Cirrhosis

**DOI:** 10.7759/cureus.61626

**Published:** 2024-06-03

**Authors:** Muhammad Qaiser Panezai, Raja Taha Yaseen, Shoaib Ahmed Khan, Ghazi Abrar, Muhammad Ali Khalid, Muhammad Manzoor Ul Haque, Ghulamullah Lail, Danish Kumar, Syed Mudassir Laeeq, Nasir Hassan Luck

**Affiliations:** 1 Department of Hepatogastroenterology, Sindh Institute of Urology and Transplantation, Karachi, PAK; 2 Department of Medicine/Gastroentelogy, Jinnah Medical & Dental College, Karachi, PAK

**Keywords:** factors, cirrhosis, frailty, predictors, hepatitis c

## Abstract

Introduction

Frailty is noticed in a large number of cirrhotic patients with advanced liver disease. Frailty not only disposes cirrhotic patients to increased rates of decompensation and hospitalization but also leads to prolonged hospital stay and increased psychological and social impact, resulting in the delisting of these patients from the transplant list. Therefore, our aim was to identify the factors that are independent predictors of frailty in patients with liver cirrhosis.

Methods

This cross-sectional study was carried out at the Department of Hepatogastroenterology, Sindh Institute of Urology and Transplantation, Karachi, Pakistan, from March 1, 2022, to August 31, 2022. All the patients diagnosed with liver cirrhosis and aged 18-70 years were included in the study. The excluded patients comprised those with disorders that over-estimate frailty such as cardiopulmonary disease and hepatocellular carcinoma. The measurement of the Liver Frailty Index (LFI) was done using the hand grip strength method, timed chair stands, and balance testing. Patients with LFI >4.5 were considered frail. All data was entered and analyzed using IBM SPSS Statistics for Windows, Version 22.0 (Released 2013; IBM Corp., Armonk, New York, United States). Continuous variables were analyzed using the student-t test while categorical variables were analyzed using the chi-square test. Variables with significance on univariate analysis then underwent multivariate analysis to identify the independent predictors of frailty in cirrhotic patients. A p-value < 0.05 was considered statistically significant.

Results

A total of 132 patients were included in the study. Out of them, 89 (67.4%) were males. On assessment, 51 (38.6%) patients were frail on presentation. On univariate analysis, female gender, advanced age, raised total leucocyte count, increased percentage of neutrophils on peripheral smear, raised serum creatinine, raised total bilirubin, raised prothrombin time, high Child Turcotte Pugh (CTP) score, and high model for end-stage liver disease along with low hemoglobin and low serum albumin levels were statistically significantly associated with frailty in cirrhosis. On multivariate analysis, female gender, age >40 years, CTP>B7, Hemoglobin <10g/dl, and neutrophils >60% on peripheral smear were independent predictors of liver frailty in cirrhotic patients.

Conclusion

Female gender, advanced age, increased neutrophils on peripheral smear, decreased hemoglobin along with increased degree of liver dysfunction were independent predictors of increased frailty in patients with chronic liver disease.

## Introduction

Cirrhosis is the ultimate result of sustained liver damage as a result of viral infections, autoimmune disorders, metabolic diseases, alcoholic steatohepatitis, non-alcoholic steatohepatitis, and cholestasis [1]. Cirrhosis is characterized by fibrous bands and nodule formation on analysis of liver tissue [2]. Ascites, portosystemic encephalopathy, variceal bleeding, hepatocellular carcinoma, and various other complications can develop as a result of cirrhosis [3].

Frailty is defined as a decreased physiological reserve and increased susceptibility to adverse outcomes due to various stressors like infections and ischemic events [4]. Frailty is a common feature of the cirrhotic population, documented in 17-43% of cirrhotic patients [5]. Different methods are being employed to measure frailty which include the Fried Frailty Index, short performance battery, and Liver Frailty Index (LFI). Among these scores, LFI is time-saving, easy to perform, and has equivalent performance when compared to the others [6].

Those cirrhotic patients who are frail have greater risks of decompensation, hospitalization, mortality, and post-transplant complications [7]. Frailty is a dynamic process and blocking further deterioration in frailty may impede the adverse complication of cirrhosis. Improvement in frailty results in improved overall survival, thus enhancing the quality of life in cirrhotic patients [8].

To the best of our knowledge, data regarding the predictors of frailty in cirrhotic patients is scarce. Thus, this study will help us identify factors predictive of frailty and this is important as managing these predictive factors will help us in improving the quality of life in cirrhotic patients. Therefore, our aim was to identify the non-invasive predictors of frailty in patients with liver cirrhosis.

This article was previously presented as a poster at the 2023 Asian Pacific Digestive Week Federation (APDWF) annual meeting on December 9, 2023.

## Materials and methods

This cross-sectional study was carried out at the Department of Hepatogastroenterology, Sindh Institute of Urology and Transplantation (SIUT), Karachi, Pakistan, from March 1, 2022, to August 31, 2022, after the approval from the Ethical Review Committee, SIUT (approval number: 412). After obtaining informed consent, all the patients of both genders diagnosed with liver cirrhosis on the basis of ultrasound abdomen, and aged 18-70 years were enrolled in this study. Patients with a prior history of disorders that overestimated frailty including those with pulmonary or cardiovascular disease or those with disorders causing restricted joint movements like osteoarthritis, rheumatoid arthritis, or connective tissue disorders, and those with impaired mentation or history of malignancy were excluded. LFI was assessed by means of the hand grip strength method, timed chair stands, and balance testing. Patients with LFI >4.5 were considered frail [9,10].

Statistical analysis

For the data analysis, IBM SPSS Statistics for Windows, Version 22.0 (Released 2013; IBM Corp., Armonk, New York, United States) was utilized. Mean±SD was used to express continuous variables while frequencies and percentages were used for the expression of categorical variables. Outcomes were measured in terms of frailty (LFI>4.5). Student T-test was used for the comparison of variables between frail and non-frail groups while Chi-square test was used for categorical variables. Statistically significant variables on univariate analysis then underwent multivariate Cox-regression analysis to identify the independent predictors of frailty in cirrhotic patients. A p-value < 0.05 was considered statistically significant.

## Results

A total of 132 patients were included in the study and the baseline characteristics are given in Table 1. The mean age was 47±12.3 years. Out of them, 89 (67.4%) were males while 43 (32.6%) were females. Hepatitis C was the most common etiology of chronic liver disease observed in 69 (52.3%) patients followed by hepatitis B in 43 (33.5%) patients, autoimmune hepatitis in seven (5.3%), and non-alcoholic fatty liver disease in four (3%), respectively. Twenty-one (15.9%) patients had a history of one or more episodes of hepatic encephalopathy while a previous history of secondary bacterial peritonitis was noted in eight (6.1%) patients. A history of esophageal varices was noted in 54 (40.9%) patients while there was a history of variceal band ligation previously in 16 (12%) patients. A history of hepato-renal syndrome was also observed in 12 (9%) patients. On assessment, 51 (38.6%) patients were frail at the time of presentation. Hospital admission was required in 56 (42.4%) patients due to liver-related complications.

**Table 1:** Baseline characteristics of the patients included in the study (N=132) CTP: Child Turcotte-Pugh, MELD-Na: Model for End-stage Liver Disease-sodium

Study population		Values
Age (years), mean±SD		46±13
Gender, n (%)	Male	89 (67.4)
	Female	43 (32.6)
Secondary Bacterial Peritonitis, n (%)	Present	8 (6.1)
	Absent	124 (93.9)
History of Encephalopathy, n (%)	Present	21 (15.9)
	Absent	111 (84.1)
Presence of Esophageal Varices, n (%)	Present	54 (40.9%)
	Absent	78 (59.1)
History of Variceal Band ligation, n (%)	Present	16 (12)
	Absent	116 (88)
Presence of Hepatorenal Syndrome, n (%)	Present	12 (9)
	Absent	120 (91)
Frailty (LFI>4.5), n (%)	Present	51 (38.6)
	Absent	81 (61.4)
Hospital Admission, n (%)	Required	56 (42.4)
	Not Required	76 (57.6)
Hemoglobin (g/dL), mean±SD		10.2±2
Total Leucocyte Count (x10^9^/L), mean±SD		5.5±2.8
Platelet Count (x10^9^/L), mean±SD		99.7±65.9
Neutrophil count (%), mean±SD		57.7±18.2
Lymphocyte count (%), mean±SD		21.7±11.2
Serum creatinine (mg/dl), mean±SD		1±0.8
Sodium (mEq/L), mean±SD		137±5.3
Total Bilirubin (mg/dl), mean±SD		2.8±4
Serum Albumin (g/dl), mean±SD		2.9±0.74
CTP, mean±SD		8.1±2.1
MELD-Na, mean±SD		14.8±6.5
Hand Grip Strength (kg), mean±SD		20.4±14
Balance Testing (sec), mean±SD		9.9±0.87
Timed Chair Stands (sec), mean±SD		14.7±10.3
Liver Frailty Index, mean±SD		4.1±0.83

Mean hemoglobin was of 10.2±2 (g%), total leucocyte count of 5.5±2.8 (x10^9^/L), platelet count of 99.7±65.9 (x10^9^/L), neutrophil count of 57.7±18.2 (%), lymphocyte count of 21.7±11.2 (%), total bilirubin of 2.8±4 (mg/dl), serum creatinine of 1±0.8 (mg/dl), prothrombin time of 13.6±3.1 (sec), CTP score of 8.1±2.1, MELD-sodium (MELD-Na) of 14.8±6.5, and serum albumin of 2.9±0.72 (g/dl) (Table 1). Mean hand grip strength was 20.4+14 kg, balance testing was 9.9+0.87 seconds and timed chair stand was 14.7+10.3 seconds. Using these variables, frailty was measured using the LFI, and the mean LFI was 4.1+0.83.

On comparative analysis, female gender, advanced age, increased total leucocyte count (TLC), percentage of neutrophils on peripheral smear, serum creatinine, total bilirubin, prothrombin time, Child Turcotte-Pugh (CTP) score, and MELD-Na score while decreased hemoglobin and serum albumin were associated with increased frailty in patients with cirrhosis (Table 2). However, no significant difference was observed in terms of peripheral lymphocyte count between the frail and non-frail population.

**Table 2:** Comparison of different variables in predicting frailty in cirrhotic patients LFI: Liver Frailty Index; CTP: Child Turcotte-Pugh; MELD-Na: Model for End-stage Liver Disease-sodium; PT: prothrombin time

Variable		LFI>4.5 (N=51)	LFI<4.5 (N=81)	p-value
Gender, n (%)	Male	26 (50.4)	63 (77.8)	0.001
	Female	25 (49.6)	18 (22.2)	
Hemoglobin (g/dl), mean±SD		8.8±1.7	10.4±2.25	0.001
Total Leucocyte Count (x10^9^/L), mean±SD		6.7±3.9	5.2±2.1	0.001
Neutrophil Count (%), mean±SD		62.8±22.2	54.3±17.9	0.018
Lymphocyte Count (%), mean±SD		16.9±11.7	21.3±9.9	0.155
Age (years), mean±SD		49.8±13.7	45.7 ±9.2	0.001
CTP score, mean±SD		9.5±1.98	7.9±1.74	<0.001
MELD-Na score, mean±SD		19.7±8.6	13.3±3.91	<0.001
Serum Albumin (g/dl), mean±SD		2.5±0.56	2.9±0.71	<0.001
Total Bilirubin (mg/dl), mean±SD		5.5±7.7	1.9±1.34	0.003
PT (sec), mean±SD		15.4±4.2	13.1±2.23	0.002
Serum Creatinine (mg/dl), mean±SD		1.3±1.14	0.9±0.45	0.001
Sodium (mEq/L), mean±SD		132±7.3	138 ±3.17	0.002

On multivariate analysis, female gender, age >40 years, CTP>B7, hemoglobin of <10g/dl, and neutrophils >60% on peripheral smear were independent predictors of liver frailty in cirrhotic patients (Table 3).

**Table 3:** Multivariate cox-regression analysis showing independent predictors of frailty in patients with liver cirrhosis CTP: Child Turcotte-Pugh

Variable	p-value	Odds ratio	95%CI	
			Lower Limit	Upper Limit
Female Gender	0.003	5.85	1.85	18.9
Age >40 years	<0.001	9.912	2.8	35.12
CTP >7	0.044	4.143	1.04	16.5
Hemoglobin <10 g/dl	0.002	4.923	1.82	13.3
Neutrophill >60%	<0.001	7.93	2.7	23.6

## Discussion

Frailty is a dynamic process and quality of life can be improved by impeding further decline in frailty [11]. Frail patients have an increased risk of disease progression, unplanned hospitalization, and death in both compensated and decompensated cirrhosis as compared to non-frail patients [12]. The main purpose of this study was to evaluate the factors that can have an impact on frailty. So, in this study, we came to know that higher CTP scores, advanced age, low hemoglobin (< 10g/dl), and higher neutrophil percentage on different counts of leucocytes are the factors that have an effect on frailty.

Previously, it was known that only age had an impact on frailty and was linked with adverse outcomes in the general population [13]. However, in our study we have also identified reversible factors like higher CTP score, low hemoglobin, and raised neutrophil percentage, to be directly related to an increase in frailty. Correcting the modifiable factors like anemia and increased neutrophil percentage (mostly seen in infectious states) will ensure improvement in overall quality of life, increase life expectancy, and decrease the risk of disease progression and prolonged hospitalization in the cirrhotic population. Cirrhotic patients are very vulnerable and recurrent infections as depicted by raised neutrophils and low exercise capacity due to underlying disease reinforced by anemia can further lead to a decline in physiological reserve and result in frailty. Similarly, elder patients are at risk of frailty, and targeting this group of the cirrhotic population and taking measures to slow down the further decline of performance status may also increase their survival and improve their quality of life.

 Moreover, frailty in this study was assessed by the LFI. LFI is an objective tool, more specific to liver-related diseases as compared to other frailty parameters i.e. Fried Frailty Index (FFI), clinical frailty scale, short physical performance battery (SPPB), easy to perform, and time-saving with good inter-observer reproducibility [6]. LFI captures the impact of declining liver status on extrahepatic organs as manifested by malnutrition, wasting, and functional impairment [14]. Studies have shown that LFI is correlated to MELD-Na and it not only improves the efficiency of MELD-Na while predicting short-term mortality in cirrhosis but also enhances subjective analysis of patient overall health status [9,15].

Our study will help to target a specific group of the cirrhosis population and take measures in order to save them from developing frailty in the future and improve their survival and quality of life.

Frailty is a well-known phenomenon and has many ramifications in cirrhosis [16,17]. However, little work has been done to identify predictors of frailty. Without knowing the real culprits leading to frailty, this lethal process cannot be managed. Thus, this study will open gates for further research regarding this topic and may lead to better and prolonged life for patients with cirrhosis.

We acknowledge some limitations in our study. Firstly, this was a single-center study. Secondly, we did not segregate the patients into compensated and decompensated groups. As we all know, frailty is a dynamic process and should be followed with time. In our study, we did not follow our patients and hence we consider it as one of the shortcomings of our study. However, one of the major strengths of our study was that it was a pioneer study revealing the predictors of frailty in cirrhosis and such a study has not been carried out previously, to the best of our knowledge, especially in this part of the world.

## Conclusions

Frailty has an impact in cirrhosis and is associated with increased morbidity and prolonged hospitalization in this population. Factors predicting increase risk of frailty were studied and female gender, advanced age, increased neutrophils on peripheral smear along with decreased hemoglobin and increased degree of liver dysfunction were the factors that were independent predictors of frailty in cirrhotic patients. Managing these reversible factors of frailty, i.e. raised total leucocyte count and neutrophil percentage, will not only improve the prognosis in these patients but will also result in improved overall quality of life in this population. For the validation of our results and in order to make these results widely acceptable, multi-centered studies with large sample sizes are required.
